# Depression and Risk of Mortality in People with Diabetes Mellitus: A Systematic Review and Meta-Analysis

**DOI:** 10.1371/journal.pone.0057058

**Published:** 2013-03-05

**Authors:** Fleur E. P. van Dooren, Giesje Nefs, Miranda T. Schram, Frans R. J. Verhey, Johan Denollet, François Pouwer

**Affiliations:** 1 CoRPS – Center of Research on Psychology in Somatic Diseases, Department of Medical and Clinical Psychology, Tilburg University, Tilburg, The Netherlands; 2 MHeNS – Alzheimer Centre Limburg, School for Mental Health and Neuroscience, Maastricht University, Maastricht, The Netherlands; 3 MUMC – Maastricht University Medical Center, Department of Medicine, Maastricht University, Maastricht, The Netherlands; Charité University Medicine Berlin, Germany

## Abstract

**Objective:**

To examine the association between depression and all-cause and cardiovascular mortality in people with diabetes by systematically reviewing the literature and carrying out a meta-analysis of relevant longitudinal studies.

**Research Design and Methods:**

PUBMED and PSYCINFO were searched for articles assessing mortality risk associated with depression in diabetes up until August 16, 2012. The pooled hazard ratios were calculated using random-effects models.

**Results:**

Sixteen studies met the inclusion criteria, which were pooled in an overall all-cause mortality estimate, and five in a cardiovascular mortality estimate. After adjustment for demographic variables and micro- and macrovascular complications, depression was associated with an increased risk of all-cause mortality (HR = 1.46, 95% CI = 1.29–1.66), and cardiovascular mortality (HR = 1.39, 95% CI = 1.11–1.73). Heterogeneity across studies was high for all-cause mortality and relatively low for cardiovascular mortality, with an I-squared of respectively 78.6% and 39.6%. Subgroup analyses showed that the association between depression and mortality not significantly change when excluding three articles presenting odds ratios, yet this decreased heterogeneity substantially (HR = 1.49, 95% CI = 1.39–1.61, I-squared = 15.1%). A comparison between type 1 and type 2 diabetes could not be undertaken, as only one study reported on type 1 diabetes specifically.

**Conclusions:**

Depression is associated with an almost 1.5-fold increased risk of mortality in people with diabetes. Research should focus on both cardiovascular and non-cardiovascular causes of death associated with depression, and determine the underlying behavioral and physiological mechanisms that may explain this association.

## Introduction

Depression is common in people with diabetes, affecting approximately 20% of the patients [1], [2]. Two meta-analyses revealed that people with type 2 diabetes mellitus are 15–24% more likely to develop depression compared to people without diabetes [3], [4]. Furthermore, individuals with previously diagnosed diabetes have an increased risk of depression relative to those with impaired glucose metabolism or undiagnosed diabetes [5]. Depressed individuals with diabetes report lower quality of life [6] have higher HbA_1c_ levels, indicating suboptimal glycemic control [7] and are characterized by poor self-care behavior that may contribute to suboptimal glycemic control [8]. They demonstrate lower levels of physical activity [9], have more negative appraisals of insulin therapy [10], are likely to be less adherent to the prescribed treatment regimen and have less healthy eating behaviors [8].

Several longitudinal epidemiological studies concluded that the combination of diabetes mellitus and depression is associated with higher mortality rates [11], [12], [13]. For example, Black et al. [11] demonstrated that people with comorbid depression and diabetes have a higher risk of developing diabetes-related complications and also mortality, than those with depression or diabetes alone or without either condition.

Lin and colleagues [14] showed that people with type 2 diabetes and comorbid depression had a 36% increased risk of developing microvascular complications such as end-stage renal disease, low vision or blindness, retinopathy, foot ulcers or amputations, compared to individuals with diabetes without depression. Furthermore, a 25% higher risk of developing macrovascular complications, such as myocardial infarction or stroke, was established.

However, the research question whether depression in diabetes is associated with an increased mortality risk has not been the subject of a systematic review and meta-analysis. Therefore, the objective of the present paper was to examine whether depression increases the risk for all-cause and cardiovascular mortality in people with diabetes, both by (a) reviewing the literature in a systematic way and (b) carrying out a meta-analysis of longitudinal studies on this subject.

## Methods

### Search Strategy

Literature searches were conducted through August 16, 2012 using the electronic databases PUBMED and PSYCINFO. The following search terms were applied: “diabetes” (title/abstract) or the medical subject headings (MeSH) “Diabetes Mellitus”, in combination with “depression” (title/abstract) or “depressive disorder” (title/abstract) or “depressive” (title/abstract) or the MeSH terms “Depression” or “Depressive disorder”, combined with “mortality” (title/abstract) or “death” (title/abstract) or the MeSH terms “Mortality” or “Death” or “Diabetes Complications/mortality”, combined with “cohort study” (title/abstract) or “longitudinal” (title/abstract) or “prospective” (title/abstract) or “cohort” (title/abstract) or the MeSH term “Cohort Studies”. No restrictions were used. Additionally, reference lists of included articles were screened by the first author (FvD) to detect complementary articles which met the selection criteria.

### Study Selection

Two authors (FvD, GN) independently evaluated the articles for eligibility. Studies that met the following criteria were included: 1) the study design was longitudinal, including both retrospective as prospective studies, 2) the study population included people diagnosed with diabetes (clinical or self-report) either as total sample or subgroup, 3) the outcome variable was mortality, 4) the association between baseline depression (yes/no, clinical diagnosis or self-report) and mortality during follow up was analysed. Studies presenting clinical trials meeting these inclusion criteria were not included in the meta-analysis, considering these studies describe the effect of a treatment in people with diabetes and depression on mortality and not the effect of depression on mortality in people with diabetes.

**Figure 1:**
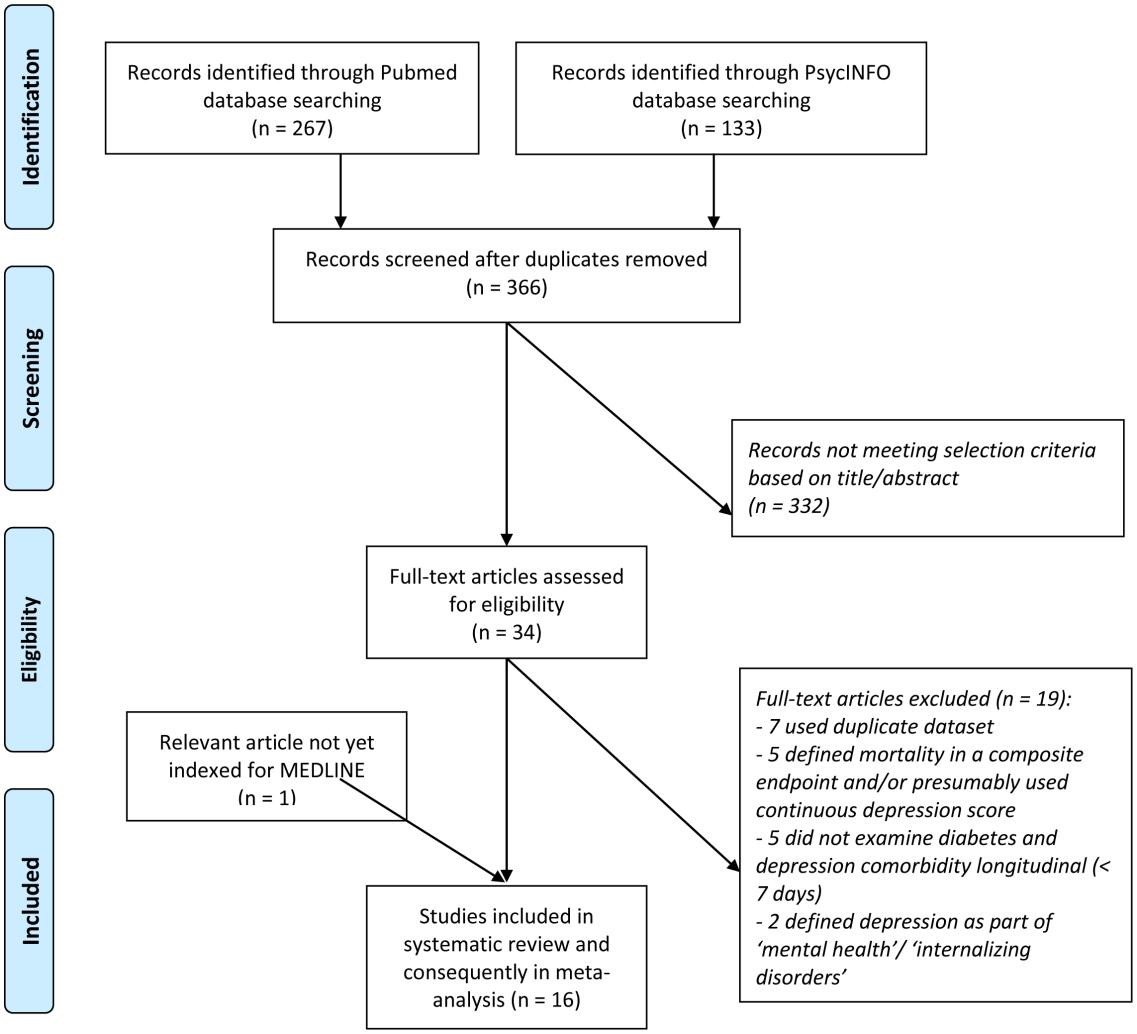
Flow diagram for selection of studies.

All discrepancies were resolved after rechecking the source papers and further discussion among both authors, and consultation of a third co-author (FP) where needed, with full consensus before inclusion. Regarding multiple reports on the same dataset, only one report was included in the meta-analysis, based upon population size (largest sample size), aim of the article (with mortality as the main end point) and primary analysis (as compared to secondary analysis). Three corresponding authors were contacted for additional information, but we did not receive further information. No restriction on type of language was placed. Figure 1 depicts the process of article selection by means of a flow diagram.

### Endpoint

The endpoint was all-cause mortality and, where available, cardiovascular mortality.

### Data Extraction and Statistical Analysis

The results of the data extraction were summarized in a systematic manner including the following information: first author name, publication year, country of study, length of follow-up, study design and sample size (mean age, % female, type of diabetes), number of depression cases, assessment method of diabetes/depression/mortality (Table 1), hazard ratios (HRs) with corresponding 95% CI and multivariable adjustment (Table 2).

**Table 1:** Overview of all included studies, sorted on publication date in descending order.

Author, year, country	Follow-up (years)	Study design and population	Number of participants (mean age, sex, type of diabetes)	Number of depression (%)	Diabetes assessment	Depression assessment	Mortality assessment
Sullivan et al. [15] 2012, USA+Canada	4.7	Multicenter (77 clinical centers) randomized controlled treatment trial testing independent effects of two strategies of control of blood glucose, blood pressure and lipids on cerebrovascular disease in people with type 2 diabetes, in USA and Canada(Action to Control Cardiovascular Risk in Diabetes Health-Related Quality of Life substudy/ACCORD HRQL)	2,053 (62 years, 40% female, type 2)	624 (31%)	American Diabetes Association criteria (1997)	PHQ-9≥10	Not reported
Bot et al. [21] 2012, The Netherlands	6.2	Multicenter cohort studies of myocardial infarction patients, recruited from 14 hospitals located in different parts of The Netherlands (subgroup with diabetes)(Depression and Myocardial Infarction Study and the Myocardial Infarction and Depression-Intervention Trial/DepreMI and MIND-IT)	330 (65 years, 30% female, type of diabetes not specified)	106 (32%)	Self-reported diagnosis at admission, which was verified by medical chart, or new diagnosis at discharge requiring medication	BDI ≥10	Statistics Netherlands/Municipal Personal Records Database, ICD-10 codes in mortality records
Winkley et al. [47] 2012, United Kingdom	5	Population-based prospective cohort of adults with diabetes and presenting with their first (baseline) foot ulcer, recruited from hospital foot and community chiropody clinics in south London, UK	253 (62 years, 36% female, type 1 and type 2 [83%])	82 (32%)	WHO criteria	SCAN 2.1	UK Central Register Office
Ahola et al. [16] 2012, Finland	9	Large national multicentre prospective study including people with type 1 diabetes, in Finland(Finnish Diabetic Nephropathy Study/FinnDiane)	4,174 (39 years, 49% female, type 1)	313 (8%)	Onset of diabetes before the age of 35 years, permanent insulin treatment initiated within 1 year of diagnosis and C-peptide negativity.	Purchase of antidepressant agents within 1 year prior to baseline	Finnish Cause of Death Register
Scherrer et al. [13] 2011, USA	7	A cohort of people free of cardiovascular disease at baseline, selected from the Veterans Administration electronic medical records (subgroup with diabetes)	53,632 (56 years/12% female for total sample, type 2)	12,679 (24%)	ICD-9-CM codes or a prescription for type 2 diabetes medication	ICD-9-CM codes	Veterans Administration Vital Status File
Pan et al. [19] 2011, USA	6	Prospective cohort study of female nurses residing in eleven states of the USA (subgroup with diabetes) (Nurses’ Health Study)	4,873 (68 years, 100% female, type 2)	1,000 (21%)	Self-reported diabetes, followed by ≥1 criteria reported on the diabetes questionnaire according to the National Diabetes Data Group	Self-reported physician-diagnosed depression, use of antidepressant medications, or self-reported symptoms of depression (MHI-5; ≤52)	Reports from next of kin, postal authorities, the National Death Index, copies of death certificates and medical records
Pieper et al. [20] 2011, Germany	3.5	Prospective clinical epidemiologic study in individuals recruited from general practices in Germany (subgroup with diabetes)(DETECT Study)	1,141 (67 years, 52% female, type 2)	165 (14%)	Clinical judgment of a doctor, use of diabetes medication, fasting blood glucose	DSQ ≥8	Doctor report, death certificates
Iversen et al. [48] 2009, Norway	10	Population-based sample of adults from a well-defined geographic area, Nord- Trøndelag County (subgroup with diabetes)(Nord- Trøndelag Health Study/HUNT 2)	1,494 (66 years, 50% female, type 1 and type 2 [82%])	258 (17%)	Self-report derived from 1 question, *“Do you have or have you had diabetes?”,* followed by non-fasting and fasting blood glucose samples	HADS-D ≥8	Norwegian Causes of Death Registry (ICD-10 codes)
Lin et al. [17] 2009, USA	5	Prospective cohort study of primary care patients with type 2 diabetes at Group Health Cooperative (mixed-model prepaid health plan) in Washington state, USA(Pathways Epidemiologic Follow-up Study)	4,184 (64 years, 49% female, type 2)	850 (20%)	In preceding 12 months: filled prescription for diabetes medication, or 2 fasting plasma glucose levels ≥126 mg/dL, or 2 random plasma glucose levels ≥200 mg/dL, or 2 outpatient diagnoses of diabetes or any inpatient diagnosis	PHQ-9***	Death registry files of Washington state, telephone survey, review of medical records, autopsy reports and death certificate data
Katon et al. [49] 2008, USA	2	Cohort of older adults with diabetes, who are Medicare FFS beneficiaries in nine counties of the state of Florida, USA	10,704 (76 years, 44% female, type of diabetes not specified)	1,657 (15%)	ICD-9 codes or Diagnosis Related Group codes	ICD-9 codes (sensitivity analysis: ICD-9 codes or PHQ-2≥3 or self-report of antidepressant medication use)	Medicare claims and eligibility files, information from telephone contact with participant’s family
Richardson et al. [50] 2008, USA	10	Cohort study of male veterans with type 2 diabetes, from a Veterans Affairs facility in the southeastern USA	14,500 (62 years, 0% female, type 2)	806 (6%)	Having ≥2 ICD-9 codes for diabetes and ≥2 visits each year since diagnosis	ICD-9 codes	Beneficiary Identification and Record Locations files ( = a national database of veterans who applied for death benefits)
Bruce et al. [51] 2005, Australia	7.8	Prospective observational study of people with diabetes from a postcode-defined community in Fremantle, Western Australia(Fremantle Diabetes Study/FDS)	1,273 (64 years, 51% female, type 2)	401 (32%)	Clinically defined: managed with diet and/or oral hypoglycemic agents regardless of age at diagnosis; (ii) ≥60 years at diagnosis whatever their treatment history; and (iii) diagnosed between 40–60 years of age and taking insulin at the time of study entry, but whose first treatment was not insulin and associated with BMI >30 kg/m^2^	≥2 GHS symptoms of depression	State registry records of Western Australia
Egede et al. [18] 2005, USA	8	Population-based follow-up study of a national probability sample of the civilian non-institutionalized population of the USA (subgroup with diabetes)(National Health and Nutrition Examination Survey I Epidemiologic Follow-up Study/NHEFS)	715 (63 years, 62% female, type of diabetes not specified)	262 (37%)	Self-report based on 1 survey question *(Have you ever been told by a doctor that you have diabetes)?)*	CES-D ≥16	Proxy interviews, health care facility records, death certificates, National Death Index, ICD-9 codes
Kuo et al. [25] 2004, USA	2	Longitudinal cohort of people with no disability in activities of daily living at baseline, enrolled in Medicare Managed Care services (subgroup with diabetes)(Medicare Health Outcomes Survey/HOS)	8,949 (≥65 years, 47% female, type of diabetes not specified)	1,915 (21%)	Self-report	Self-report based on 3 questions: *1) “In the past year, have you had ≥2 weeks during which you felt sad, blue, or depressed, or when you lost interest or pleasure in things that you usually cared about or enjoyed?”; 2) “In the past year, have you felt depressed or sad much of the time?”; 3) “Have you ever had ≥2 years in your life when you felt depressed or sad most days, even if you felt okay sometimes?”*	Cohort 1 Analytic Public Use File published by Center for Medicare and Medicaid Services, Health Services Advisory Group
Black et al. [11] 2003, USA	7	Longitudinal population-based study of Mexican Americans residing in the southwestern USA (subgroup with diabetes)(Hispanic Established Population for the Epidemiologic Study of the Elderly/EPESE)	636 (≥65 years, 59% female for total sample, type 2)	188 (30%)	Self-report based on 1 interview question *(Has a doctor ever told you that you have diabetes? Type 1/2?)*	CES-D ≥16	Assessed at each follow-up interview, death certificates
Rosenthal et al. [22] 1998, USA	3	A prospective study of older people with diabetes	135 (70 years, 4% female, type of diabetes not specified)	45 (33%)	Previous diabetes management in other clinics	Yesavage Depression Inventory Score >9	Direct medical history, review of medical records and death certificates

•CES-D = Center for Epidemiologic Studies Depression Scale.

•BDI = Beck Depression Inventory.

•DSQ = Depression Screening Questionnaire.

•GHS = General Health Status questionnaire.

•HADS-D = Hospital Anxiety and Depression Scale – depression subscale.

•ICD-9/10 (-CM) = International Classification of Diseases 9/10 (-Clinical Modification).

•MHI-5 = Mental Health Inventory 5-items, a subscale of the 36-item Short-Form Health Survey.

•PHQ-9/2 = Patient Health Questionnaire 9 items/2 items.

•Scan 2.1 = Schedules for Clinical Assessment in Neuropsychiatry 2.1.

•WHO = World Health Organisation.

•* Minor depression: ≥1 core symptom (depressed mood or anhedonia) +2–4 depressive symptoms for more than half the time, for ≥2 last weeks.

•** Major depression: ≥1 core symptom (depressed mood or anhedonia)+≥5 depressive symptoms for more than half the time, for ≥2 last weeks.

**Table 2:** All-cause and cardiovascular mortality risk in people with diabetes and depression, compared to people with diabetes without depression.

Author, year	Follow-up (years)	Selected estimate (95% CI) (all-cause)	Selected estimate (95% CI) (cardiovascular)	Adjustment variables
Sullivan et al. [15] 2012	4.7	1.76 (1.12–2.78)		Assignment to one of eight study intervention arms, primary/secondary prevention status, age, sex, race/ethnicity, BMI, weight, waist circumference, duration of diabetes, blood pressure, triglycerides, LDL and HDL cholesterol, serum creatinine, HbA_1c_, fasting glucose, presence of microvascular complications, blood pressure and lipid medications, education, smoking, alcohol consumption, living alone
Bot et al. [21] 2012	6.2	2.10 (1.38–3.21)	2.54 (1.32–4.89) #	Age, sex, smoking, hypertension, previous myocardial infarction, Killip class, left ventricular ejection fraction
Winkley et al. [47] 2012	5	2.09 (1.34–3.25)		Age, sex, marital status, socioeconomic status, smoking, mean HbA1c, ulcer severity
Ahola et al. [16] 2012 *	9	1.53 (1.10–2.13)		Age, diabetes duration, diastolic blood pressure, smoking, HbA1c, nephropathy
Scherrer et al. [13] 2011 *** ( = RR)	7	1.04 (0.96–1.13)		–
Pan et al. [19] 2011 ** ( = RR)	6	1.53 (1.29–1.82)	1.63 (1.19–2.22) +	Age, ethnicity, marital status, family history of diabetes and cancer, parental history of myocardial infarction, BMI, physical activity, alcohol consumption, smoking, multivitamin use, estrogen hormone use, aspirin use, hypertension, hypercholesterolemia, heart disease, stroke, cancer
Pieper et al. [20] 2011 **	3.5	1.53 (0.88–2.66)		Age, gender, distribution of physicians throughout the country, waist circumference, education, profession
Iversen et al. [48] 2009	10	1.37 (1.10–1.72)		Age, sex, education, smoking, waist circumference, cardiovascular disease, history of foot ulcers
Lin et al. [17] 2009 *	5	1.46 (1.23–1.75)	1.31 (0.99–1.73) +	Age, sex, race, education, marital status, diabetes duration, type of treatment, medical comorbidity, hypertension
Katon et al. [49] 2008	2	1.36 (1.16–1.59)		Age, gender, race/ethnicity, Charlson score ( = comorbidity index), prior CVA, prior CVD, prior CVD procedure, prior amputation
Richardson et al. [50] 2008	10	1.6 (1.3–1.8)		Age, race/ethnicity, marital status, employment status and comorbidity (CHD, hypertension, stroke and cancer)
Bruce et al. [51] 2005	7.8	1.21 (0.95–1.55)	1.15 (0.80–1.68) #	Age, sex, ethnicity, HbA1c, BMI, diabetes duration, smoking, physical activity, blood pressure-lowering therapy, CHD, CVD, albumin/creatinine ratio, retinopathy, neuropathy
Egede et al. [18] 2005	8	1.33 (1.02–1.74)	1.07 (0.67–1.71) $	Age, sex, race/ethnicity, poverty: income ratio, education, marital status, smoking, physical activity, BMI, aspirin use, comorbid medical conditions at baseline (cancer, hypertension, heart disease, stroke)
Kuo et al. [25] 2004 *** ( = RR)	2	0.97 (0.75–1.24)		Age, sex, marital status, education, hypertension, angina or coronary artery disease, myocardial infarction, other heart condition, stroke, COPD, arthritis, cancer, general health, social functioning
Black et al. [11] 2003 */**	7	2.08 (1.39–3.12)		Age, sex, education, acculturation, marital status
Rosenthal et al. [22] 1998 *** ( = RR)	3	4.50 (1.52–10.43)		-

* = pooled hazard ratio of two groups.

** = hazard ratio with diabetes and no depression as reference category, recalculated from four group scenario (diabetes yes/no x depression yes/no) with no diabetes/no depression as reference category.

*** = odds ratios converted to risk ratios, which then can be combined with hazard ratios in a meta-analysis as forms of relative risks.

# = cardiac mortality.

+ = cardiovascular disease mortality.

$ = coronary heart disease mortality.

•BMI = Body Mass Index.

•CVA = cerebrovascular accident.

•CVD = cardiovascular disease.

•CHD = coronary heart disease.

•COPD = chronic obstructive pulmonary disease.

Data from all studies were pooled using the program Comprehensive Meta-Analysis version 2 (Biostat, Englewood NJ, 2005). If multiple hazard ratios were presented in a given article, the estimate that most closely adjusted for demographic variables and micro- and/or macrovascular complications (e.g. retinopathy, neuropathy) was selected, to expose the independent effect of depression.

When appropriate, the meta-analysis will be performed using the fixed effects or the random effects model: in case of homogeneity (or low heterogeneity) the fixed effects model will be used. If heterogeneity is substantial (above 50%) the random effects model will be used. In two studies [11], [15] more than one measure of depression was used. Black and colleagues [11] measured depressive symptoms at baseline with the CES-D, and also used a modified version of the CIDI at two years follow up to determine whether patients suffered from a depressive disorder. Depressive symptom-scores were included, as they were measured at baseline. Sullivan et al. [15] reported minor and major depression based on the PHQ-9, the dichotomisation of PHQ-9≥10 and the continuous PHQ-9 score. As combining major and minor depression into one estimate would require an additional step, the readily available dichotomised PHQ-9 score was used.

For three studies [11], [14], [16] hazard ratios needed to be combined in order to obtain the estimate of interest (example of procedure explained in Figure S1 in Supporting Information S1). In one paper [16] the hazard ratios for men and women were separately reported. In the case of Lin et al. [17] we assembled the hazard ratio for minor and major depression into one depression hazard ratio. For Black et al. [11], we combined the group with minimal depression (CES-D = 1–15) and without any depressive symptoms (CES-D = 0) into one group (CES-D <16).

Five studies [11], [18], [19], [20], [21] used the no diabetes, non-depressed group as the reference category, while we were interested in the comparison of the two diabetes groups only (depressed versus non-depressed). Two of these studies [18], [21] performed post hoc analyses in people with diabetes only, producing the desired estimate.

For the other three studies, we used the information from the four group scenario to calculate the HR for the comparison of the depressed and non-depressed diabetes groups (example of procedure explained in Figure S2 in Supporting Information S1).

In two papers [13], [22] no hazard ratios or odds ratios were presented, so the odds ratios and confidence intervals for these studies were calculated based on the number of patients who died in each group.

Statistical heterogeneity was assessed using the I-squared statistic, which quantifies the percentage of total variation across studies due to heterogeneity rather than chance, with values of 50% or more indicating a substantial level of heterogeneity [23]. When study outcomes were heterogeneous based on this statistic, the potential influence of follow-up length, age, method of depression assessment, method of diabetes assessment, number of participants and the percentage of females included were examined. Differences in effect estimates between the subgroups were assessed by comparing the pooled effect estimates using chi-squared analysis, comparing logarithms of these estimates. Additionally, a sensitivity analysis where each study is removed one by one was done.

Potential publication bias was assessed with Egger’s test of the intercept [24]. A funnel plot was constructed by plotting the effect measure against the inverse of its standard error (see Figure S3 in Supporting Information S1. An asymmetric plot indicates a likely publication bias, and p<0.05 is considered representative of statistically significant publication bias.

## Results

A total of 400 potentially relevant articles were retrieved by the database searches (Figure 1). From this set, 34 full-text articles were assessed for eligibility. Of these, 15 articles met our inclusion criteria and were included in the systematic review. Three of these articles [13], [22], [25] used a different measure of effect size (odds ratio) which cannot be pooled in a meta-analysis with hazard ratios [26]. However, odds ratios can be converted to risk ratios, which then can be combined with hazard ratios in a meta-analysis as forms of relative risks [27].

One additional relevant article that was not indexed for MEDLINE yet, was included in both the review and meta-analysis [15]. The characteristics and extracted data of the 16 articles are presented in Table 1, and the hazard ratios with corresponding 95% confidence intervals and covariates used for analysis in Table 2.

### All-cause Mortality

Sixteen studies were included in the meta-analysis, comprising 109046 individuals with diabetes and including 21443 (19.7%) with comorbid depression. The follow-up periods of included studies ranged from 2–10 years, with a mean follow-up of 6 years. The mean age at baseline ranged from 62 to 76 years, with exception of the only article focusing on type 1 diabetes, with a mean age of 39 years at baseline [16]. Twelve analyses (75%) showed a statistically significant association between depression and mortality in individuals with diabetes.

**Figure 2:**
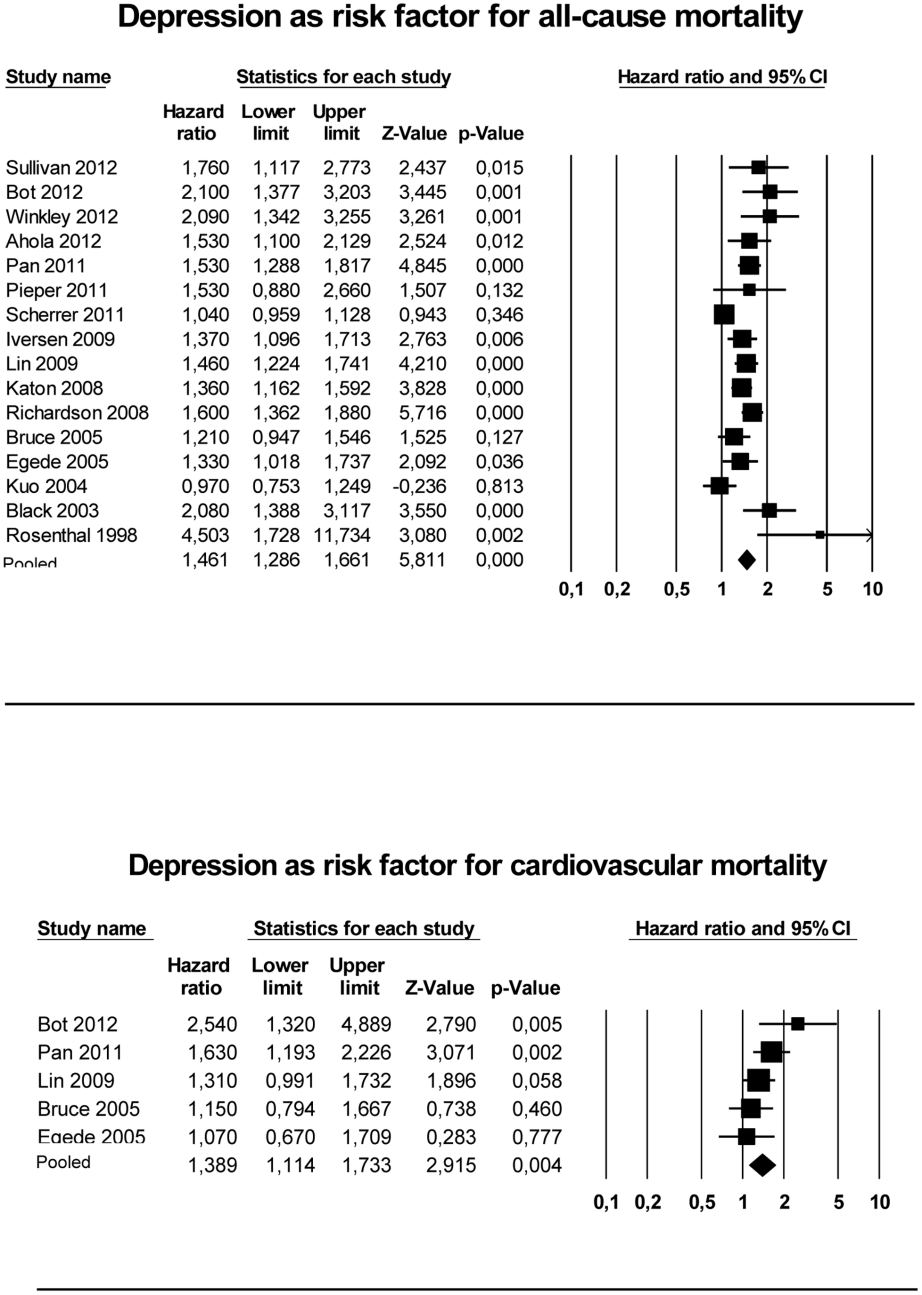
Meta-analysis of the association between depression and all-cause and cardiovascular mortality.

The pooled hazard ratio for mortality was significantly increased in patients with diabetes and depression compared with those without depression (HR 1.46, 95% CI 1.29–1.66, p<0.0001) (Figure 2). The I^2^ value was 78.6%, demonstrating high heterogeneity in the study results. After conducting subgroup analyses, there were no significant differences for follow-up length, age, method of depression assessment, method of diabetes assessment, number of participants and the percentage of females included. Only a significant difference was found after excluding the three articles presenting odds ratios (converted to risk ratios). The pooled hazard ratio of 13 studies presenting hazard ratios was 1.49, 95% CI 1.39–1.61 (p<0.0001) (in both random as fixed effects model) and an I-squared statistic of 15.1%.A funnel plot of the 16 studies (Figure S3 in Supporting Information S1) suggests evidence of publication bias and Egger’s test confirmed this finding showing significant asymmetry (p [one-tailed] <0.05).

### Cardiovascular Mortality

Five of the 16 studies, comprising 11375 individuals with diabetes and including 2619 (23%) with comorbid depression, also specifically reported on cardiovascular mortality as separate endpoint. Two examined cardiac mortality, two cardiovascular disease mortality and one coronary disease mortality. The follow-up periods ranged from 5–8 years, with a mean follow-up of 6.6 years. The mean age at baseline ranged from 63 to 68 years. Two out of 5 studies found a significant association between depression and cardiovascular mortality in people with diabetes [19], [21], and there was a trend in a third study [17]. After pooling the HRs for cardiovascular mortality, the HR was significant (HR = 1.39, 95% CI 1.11–1.73, p<0.0001) (Figure 2).

## Discussion

This meta-analysis of 16 longitudinal studies shows a positive association between depression and subsequent mortality rates in people with diabetes. Compared to those without depression, depressed individuals had a 46% increased risk for all-cause mortality. Although based on only 5 studies, our results also show a 39% increased risk for cardiovascular mortality associated with the presence of depression in diabetes.

Previous meta-analyses have also found positive associations between depression and mortality rates in the general population (RR = 1.81, 95% CI 1.58–2.07) [28] and in patients with established heart disease (OR = 2.38, 1.76–3.22 and OR = 2.59, 1.77–3.77 for all-cause and cardiac mortality, respectively) [29]. The triad of depression, diabetes and cardiovascular disease is closely interrelated. Premature cardiovascular disease is the most common cause of morbidity and mortality in people with diabetes [30] and co-morbid depression appears to increase the risk of developing vascular conditions in this group [11], [15], [17]. However, depression is also common in people with established cardiovascular disease [31]. Rather than being a (in)direct causal factor, depression in diabetes may be secondary to having cardiovascular complications. It may owe its association with mortality to the increased risk of new cardiovascular events in people with established cardiovascular conditions [32]. We took this issue into account by including the risk estimates that were adjusted for existing vascular disease, and still found an almost 1.5-fold increased risk of mortality in depressed people with diabetes. Further prospective studies are needed to examine whether depression exerts a negative influence on mortality through the development of new vascular complications. These studies may also explore whether people with comorbid diabetes and depression face increased mortality risks beyond cardiovascular deaths, as suggested by recent results from the Pathways Epidemiologic Follow-up Study [17].

There are several potential behavioral or physiological mechanisms that could explain the increase of mortality for people with diabetes and depression. Depression is correlated with a decline in health-maintenance behaviors (e.g. physical activity, smoking, diet) in general [33], which is also true for people with diabetes [34]. In addition, depression is associated with several biological alterations; activation of the hypothalamic-pituitary-adrenal axis and proinflammatory cytokines, sympathic nervous system dysregulation, decrease in heart rate variability and cardiac fibrillation threshold, which can contribute to an increased risk of cardiovascular mortality, but also mortality of other causes [35].

In line with previous studies examining post-myocardial infarction depression [29] or depression in community samples [28], we did not observe a difference in mortality risk between studies assessing depression using a self-report questionnaire versus a clinical psychiatric interview. Only a minority of 30–40% of people with diabetes with an increased level of depressive symptoms suffers from clinically relevant depressive disorder [36], [37]. However, both major depression and self-reported depressive symptoms appear to be chronic/recurrent conditions in people with diabetes [38], [39], and both are associated with the development of diabetes complications [11]. Furthermore, depressive symptoms have been shown to predict the development of major depression [40].

The results need to be considered in relation to the study limitations. One important limitation in carrying out a meta-analysis is the inevitability to combine data from studies that are not equally designed. This meta-analysis included studies with differing study design and characteristics, and the results demonstrated significant heterogeneity. After conducting subgroup-analyses on follow-up length, age, method of depression assessment, method of diabetes assessment, number of participants and the percentage of females included, heterogeneity remained. However, after excluding the three articles presenting odds ratios (converted to risk ratios) the heterogeneity decreased substantially. This may be due to the fact that odds ratios and hazard ratios are different risk estimates, even after converting odds to risk ratios and combining them with relative risks [41].

In addition, the included studies often reported multiple hazard ratios, each adjusted for different covariates. To reveal the independent effect of depression on mortality we selected the hazard ratio that was most closely adjusted for both demographic and micro- and macrovascular complications. Unfortunately, these estimates sometimes also include adjustment variables through which depression may influence mortality rates, e.g. smoking, physical activity, HbA_1c._ By correcting for these potential mediators the hazard ratio can be an underestimation of the real effect of depression on mortality in people with diabetes. With respect to type of diabetes of study participants, five articles did not specify this information. Moreover, only one article reported on individuals with type 1 diabetes, and two articles reported on a combined study population of people with both type 1 and type 2 diabetes. Because type 2 diabetes is the most prevalent form of diabetes, cohort studies with patients with type 1 diabetes are scarce. Finally, we found an indication for publication bias: negative or insignificant result are often not submitted for publication by authors, or rejected by reviewers and editors. This form of bias generally results in an overestimation of the effect.

Despite these limitations several strengths should also be acknowledged. First, our meta-analysis comprises both all-cause and cardiovascular mortality. In addition, the independent effect of depression on mortality was assessed by adjusting for both demographic variables and micro- and macrovascular complications where possible.

It is still unclear whether adequate depression recognition and subsequent depression treatment can help to decrease mortality rates. Bogner et al. [42] have conducted the Prevention of Suicide in Primary Care Elderly: Collaborative Trial (PROSPECT) study that examined a care-management intervention for older primary care patients with depression. The study had a median follow-up of more than 4 year. The authors reported that depressed patients with diabetes in the intervention category were less likely to have died during the 5-year follow-up interval than depressed diabetic patients in usual care after accounting for baseline differences among patients (adjusted hazard ratio 0.49 [95% CI 0.24–0.98]). However, the statistical methods used by Bogner et al. [42] were criticized, as they may have resulted in model overfitting [43], [44]. Screening for depression in clinical practice may be a helpful first step, and should be embedded in collaborative care approaches [45]. Effective intervention strategies include cognitive behavioral therapy and treatment with antidepressant medication [46]. Given the close association of depression with suboptimal self-care behaviors [34], interventions that target behavioral mechanisms directly (e.g. coping skills training) may be of value as well.

In conclusion, the results of this meta-analysis suggest that depression is associated with a 1.5-fold increased risk of all-cause mortality in people with diabetes. Although based on only five studies, similar results were found for cardiovascular mortality. In consideration of the study limitations and strengths, we believe that a 1.5-fold increased risk of all-cause (and cardiovascular) mortality in people with diabetes is not an over- or underestimation, but could be an accurate risk estimation.

Future studies are encouraged to explore whether the association between depression and mortality is similar for people with type 1 and type 2 diabetes, and to address the behavioral or physiological pathways that may explain this association.

## Supporting Information

Checklist S1 **PRISMA Checklist**
(DOCX)

Supporting Information S1 **Online Appendix.** Figure S1, Example of calculation – combining two hazard ratios. Figure S2, Example of calculation – recalculating hazard ratios with corresponding 95% CI. Figure S3, Funnel plot meta-analysis all-cause mortality.
(DOCX)
