# Supplementary material for: Depression and Risk of Mortality in People with Diabetes Mellitus: A Systematic Review and Meta-Analysis
Source: PLoS One. 2013 Mar 5;8(3):e57058. doi: 10.1371/journal.pone.0057058 (PMC3589463; doi:10.1371/journal.pone.0057058)
Supplement: Supporting Information S1 — Online Appendix. Figure S1, Example of calculation – combining two hazard ratios. Figure S2, Example of calculation – recalculating hazard ratios with corresponding 95% CI. Figure S3, Funnel plot meta-analysis all-cause mortality. (DOCX) [file pone.0057058.s002.docx]

**Supporting Information - Online Appendix

Fig. S1 Example of calculation - combining two hazard ratios**

Let lnHR1 and lnHR2 denote the two log transformed hazard ratios and vHR1 and vHR2 the two corresponding sampling variances (the square of the standard errors).
Then let wHR1 = 1/vHR1 and wHR2 = 1/vHR2.

The combined log transformed hazard ratio is then:

lHR = (wHR1 * lnHR1 + wHR2 * lnHR2) / (wHR1 + wHR2)

and the sampling variance for lHR is given by:

vHR = 1 / (wHR1 + wHR2).

By means of the new (combined) variance, the corresponding 95% confidence interval can be calculated.

SE = √variance

log (HR) +/- 1.96 SE = upper/lower bound 95% CI

**Fig. S2 Example of calculation – recalculating hazard ratios with
 corresponding 95% CI**

**Hazard Ratio (HR)** **Confidence Interval (CI) 95%**

1) no DM2 + no depression (= original reference group) 1

2) no DM2 + depression 2.47 (1.73 - 3.52)

3) DM2 + no depression 2.57 (1.89 - 3.49)

4) DM2 + depression 3.93 (2.31 - 6.68)

To compare group 4 with group 3 (= new reference group), recalculate HR + 95%CI

**HR**  **β (=log HR)**  **β**  **new HR (=exp β)**1) 1 (ref) 🡪 0 -0. 943905898 🡪 0.056094…

2) 2.47 🡪 0.90421815 -0.039687748 🡪 0.961089…

3) 2.57 🡪 0.943905898 0 (ref) 🡪 1

4) 3.93 🡪 1.368639426 0.424733528 🡪 1.529182881

Thus, new HR:

1) 0.05609... 🡪 -

2) 0.96108... 🡪 -

3) 1 🡪 new reference group

4) 1.529182881🡪 -

Now, recalculate 95% CI (rounded numbers)

log (HR) +/- 1.96 SE = (... , ...) = 95% CI

Group 3 vs group 1 🡪

0.94 (= β original HR group 3) +/- 1.96 SE ≈ 0.64, 1.25 (= log original 95% CI)

0.94 + 1.96 SE ≈ 1.25

1.96 SE ≈ 0.31

SE ≈ 0.16 (same goes when you calculate 0.94 – 1.96 SE)

Variance = SE² ≈ 0.0256

0.94 +/- 1.96 (0.16) ≈ (... , ...)

Group 4 vs group 1 🡪
1.37 (= β original HR group 4) +/- 1.96 SE ≈ 0.84, 1.90 (= log original 95% CI)

1.37 + 1.96 SE ≈ 1.90

1.96 SE ≈ 0.53

SE ≈ 0.27 (same goes when you calculate 1.37 – 1.96 SE)

Variance = SE² ≈ 0.0729

1.37 +/- 1.96 (0.27) = (… , …)

Var (log (HR)) = Var (log (HR group 3)) + Var (log (HR group 1))

0.0256 = Var (log (HR group 3)) + Var (log (HR group 1))

0.0729 = Var (log (HR group 4)) + Var (log (HR group 1))

Min (Var (log (HR group 3)) = 0

Min (Var (log (HR group 4)) = 0

Max (Var (log (HR group 3)) = 0.0256

Max (Var (log (HR group 4)) = 0.0729

(What matters is not the size of the group itself, but the number of people in each group that experienced the event (here, died)

Events: group 3 vs group 1 = 199

group 4 vs group 1 = 139

group 1 🡪 123 ≈ 61.81% 🡪 counts for 38.19% of the variance
group 3 🡪 76 ≈ 38.19% 🡪 counts for 61.81% of the variance

group 4 🡪 16

group 1 🡪 123 ≈ 88.49% 🡪 counts for 11.51% of the variance
group 3 🡪 76

group 4 🡪 16 ≈ 11.51% 🡪 counts for 88.49% of the variance

Var group 3 = 61.81% of 0.0256 ≈ 0.0158

Var group 4 = 88.49% of 0.0729 ≈ 0.0645

Var group 3 + Var group 4 ≈ 0.0803

SE = √Var ≈ 0.2834

New HR ≈ 1.53 🡪 log 🡪 ≈ 0.4253

0.4253 + (1.96 x 0.2834) ≈ 0.9808 🡪 exp 🡪 ≈ 2.67

0.4253 - (1.96 x 0.2834) ≈ -0.1302 🡪 exp 🡪 ≈ 0.88

Thus new HR + 95% CI = 1.53 (0.88 – 2.67)

**Fig. S3 Funnel plot meta-analysis all-cause mortality**
